# Elevated *O*‐GlcNAc Levels Activate Epigenetically Repressed Genes and Delay Mouse ESC Differentiation Without Affecting Naïve to Primed Cell Transition

**DOI:** 10.1002/stem.1761

**Published:** 2014-09-15

**Authors:** Christopher M. Speakman, Tanja C.E. Domke, Wikrom Wongpaiboonwattana, Kelly Sanders, Manikhandan Mudaliar, Daan M.F. van Aalten, Geoffrey J. Barton, Marios P. Stavridis

**Affiliations:** ^1^ Division of Cancer ResearchMedical Research Institute, College of Medicine, Dentistry and Nursing, University of DundeeDundeeUnited Kingdom; ^2^ Division of Molecular MicrobiologyCollege of Life Sciences, University of DundeeDundeeUnited Kingdom; ^3^ Division of Computational BiologyCollege of Life Sciences, University of DundeeDundeeUnited Kingdom; ^4^ Glasgow PolyomicsCollege of Medical, Veterinary and Life Sciences, University of GlasgowGlasgowUnited Kingdom

**Keywords:** Embryonic stem cells, Cell differentiation, *O*‐GlcNAc, Post‐translational protein modification, Signal transduction, Oligonucleotide microarrays

## Abstract

The differentiation of mouse embryonic stem cells (ESCs) is controlled by the interaction of multiple signaling pathways, typically mediated by post‐translational protein modifications. The addition of *O*‐linked *N*‐acetylglucosamine (*O*‐GlcNAc) to serine and threonine residues of nuclear and cytoplasmic proteins is one such modification (*O*‐GlcNAcylation), whose function in ESCs is only now beginning to be elucidated. Here, we demonstrate that the specific inhibition of *O*‐GlcNAc hydrolase (Oga) causes increased levels of protein *O*‐GlcNAcylation and impairs differentiation of mouse ESCs both in serum‐free monolayer and in embryoid bodies (EBs). Use of reporter cell lines demonstrates that Oga inhibition leads to a reduction in the number of Sox1‐expressing neural progenitors generated following induction of neural differentiation as well as maintained expression of the ESC marker Oct4 (Pou5f1). In EBs, expression of mesodermal and endodermal markers is also delayed. However, the transition of naïve cells to primed pluripotency indicated by Rex1 (Zfp42), Nanog, Esrrb, and Dppa3 downregulation and Fgf5 upregulation remains unchanged. Finally, we demonstrate that increased *O*‐GlcNAcylation results in upregulation of genes normally epigenetically silenced in ESCs, supporting the emerging role for this protein modification in the regulation of histone modifications and DNA methylation. Stem Cells *2014;32:2605–2615*

## Introduction

Over the last decade there have been major advances in our understanding of the mechanisms controlling embryonic stem cell (ESC) behavior in response to the changing extracellular environment 1. These mechanisms largely involve engagement of signal transduction relays that operate by post‐translational modifications of proteins. Under standard conditions, ESCs grow as a mixture of “naïve” and “primed” cells. Signaling mediated by Erk1/2 target phosphorylation has recently been implicated in regulating the transition between these two states and initiation of differentiation 2. Reversible protein modification by addition of *O*‐linked *N*‐acetylglucosamine to serine or threonine residues (*O*‐GlcNAcylation) was first described 30 years ago 3 and occurs with similar time scales, dynamics, and stoichiometry as protein phosphorylation, with which it sometimes competes. *O*‐GlcNAcylation is found in all higher eukaryotes tested to date and has been implicated in development, epigenetic regulation, and diseases such as diabetes and Alzheimer's 4. Its addition and removal are catalyzed by one transferase (Ogt) and one hydrolase (Oga; also known as Mgea5), respectively.

To date there have been very few studies on *O*‐GlcNAc function in ESCs, although accumulating evidence suggests a critical role for this modification. Elimination of Ogt in mouse ESCs or conditional deletion in somatic cells leads to death, consistent with an essential role in all cell types 5, 6. Ogt has recently been identified as a protein partner of the essential ESC transcription factor Oct4 (Pou5f1) by three independent studies 7, 8, 9. Furthermore, Oct4 has been shown to be modified by *O*‐GlcNAc 10, and this was demonstrated to be important for regulation of a subset of its targets in ESCs and during reprogramming 11. A previous study has suggested that increased *O*‐GlcNAcylation in ESCs prevents differentiation along the cardiac lineage in spontaneously differentiating embryoid bodies (EBs) 12; however, the mechanism for this or the stage at which differentiation stalled was not determined. Ogt is a mammalian homolog of the *Drosophila super sex combs* (scx) gene, a member of the Polycomb group of transcriptional repressors 13, 14 and has established roles in gene repression 15. Furthermore, recent studies implicate *O*‐GlcNAc transferase in the regulation of the Tet epigenetic modifiers 16, 17, 18, suggesting that the *O*‐GlcNAc modification and the enzymes controlling it regulate chromatin in pluripotent cells by multiple mechanisms.

In this study, we investigate in detail the effects of increased *O*‐GlcNAcylation on neural differentiation of mouse ESCs. Our results show that Oga inhibition leads to delayed onset of differentiation although the transition of naïve cells to primed pluripotency proceeds unhindered. We also present a genome‐wide gene expression analysis of ESCs and differentiating cells treated with a highly specific Oga inhibitor, revealing that increased *O*‐GlcNAc levels control the expression of distinct groups of genes in ESCs associated with a recently described subpopulation resembling the two‐cell‐stage embryo. Upregulation of these gene sets is consistent with a disruption of the normal transcriptional repression programme operating in pluripotent cells.

## Materials and Methods

### ESC Culture and Differentiation

Mouse ESCs were cultured without feeders on 0.1% gelatin‐coated plastics in Glasgow Minimal Essential Medium with 10% serum and Leukemia Inhibitory Factor (LIF) 19 and differentiation was performed according to our previous protocol 20. The cell lines used were derivatives of E14Tg2aIV (46C 21, OCRG9 22) or Oct4GiP 23. GlcNAcstatin C (GNS) was obtained from GlycoBioChem (Dundee, UK; www.glycobiochem.com) and used at 1 µM unless otherwise specified. For flow cytometry, cells were dissociated using Accutase (Millipore, Billerica, MA; www.emdmillipore.com) and resuspended in PBS/1% bovine serum albumin (BSA) containing 1 µg/ml propidium iodide (PI; Sigma, St. Louis, MO; www.sigmaaldrich.com). Cellular debris and PI‐positive cells were excluded from analysis. For clonal analysis, 600/100 cells were plated per 10 cm dish/well of six‐well plate and cultured for 6–8 days, then fixed, and stained using an Alkaline phosphatase staining kit (Sigma).

### siRNA Induced Knockdown of Oga and Ogt

Cells were seeded into six‐well dishes (2 × 10^5^ cells/well). While still in suspension, cells were transfected with SmartPool siRNAs targeting Oga or Ogt or with a nontargeting siRNA pool (50 pmol per well, Thermo Scientific, Waltham, MA; www.thermoscientific.com) using Lipofectamine RNAiMAX (Life Technologies, Carlsbad, CA; www.lifetechnologies.com) (5 µl per well). Cells were transfected again after 24 hours.

### Western Blotting

Cells were cultured or differentiated in 9 cm dishes and treatments were for 24 hours unless otherwise specified. Lysis was performed on ice in whole‐cell lysis buffer (150 mM sodium chloride, 1.0% Nonidet P40, 50 mM Tris, pH 8.0 with 1 µM GNS, Complete protease inhibitor, and PhosStop phosphatase inhibitor cocktail tablets from Roche, Basel, Switzerland; www.roche.com). For nuclear/cytoplasmic fractionation, cells were lysed on ice in Buffer A (10 mM HEPES, 1.5 mM MgCl_2_, 10 mM KCl, 0.5 mM Dithiothreitol (DTT), 0.05% Nonidet P40 pH 7.9) for 10 minutes and centrifuged for 10 minutes at 3,000 rpm in a microfuge at 4°C. The supernatant was removed as a cytoplasmic fraction and the pellet was resuspended in Buffer B (5 mM HEPES, 1.5 mM MgCl_2_, 0.2 mM EDTA, 0.5 mM DTT, 26% glycerol [vol/vol], pH 7.9) by micropestle homogenization, NaCl added to 300 mM, and incubated on ice for 30 minutes. A final spin at 12,000*g* was performed to separate insoluble material from the nuclear fraction. Protein concentration was determined by Coomassie protein assay (Thermo Scientific) and 10–30 µg of protein was loaded per lane of Life Technologies NuPage gels and transferred to Polyvinylidene fluoride membrane (Millipore). Membranes were blocked in either 5% Milk (Marvel, Premier Foods, St. Albans, UK; www.premierfoods.co.uk) in TBST or 1%–5% BSA (Millipore) in TBST (all other chemicals from Sigma). Antibodies were incubated in blocking buffer overnight: anti‐*O*‐GlcNAc, CTD110.6 (Covance, Princeton, NJ; www.covance.com, 1/5,000), or RL‐2 (Santa Cruz Biotechnology, Dallas, TX; www.scbt.com, 1/1,000); beta actin (Abcam, Cambridge, UK; www.abcam.co.uk, 1/2,000); phosphoErk1/2 (Cell Signaling Technologies, Beverly, MA; www.cellsignal.com, 1/1,000); Gsk3α and phosphoGsk3α/β (Cell Signaling, 1/1,000); phospho‐Serine and phospho‐Threonine (Cell Signaling, 1/1,000); Oct4 (Abcam 1/1,000); Sox2 (Abcam 1/1,000). For the phospho‐kinase antibody array (R&D Systems, Minneapolis, MN; www.rndsystems.com) 500 µg of total cell lysate was used according to manufacturers instructions and the arrays were developed using LumiGLO from Cell Signaling and imaged on a Fuji LAS3000 mini scanner. Densitometry was performed with ImageJ.

### Immunoprecipitation

Whole cell lysate (500 µg) was incubated overnight with a mixture of succinylated wheat germ agglutinin‐agarose and protein A sepharose beads in the presence of antibody RL‐2 (anti‐*O*‐GlcNAc). After washing the bound proteins were eluted in 25 µl of 2× LDS loading buffer (Life Technologies), boiled for 5 minutes, and analyzed by Western blotting.

### Immunocytochemistry

Cells were fixed in 4% paraformaldehyde (PFA) for 15 minutes, permeabilized in PBS, 0.1% Tween (PBST), and incubated in PBST +5% BSA (blocking buffer). Primary antibody (goat anti‐Oct4, Santa Cruz) was diluted in blocking buffer at 1/50 and incubated overnight at 4°C. Subsequently, cells were incubated in fluorescent secondary antibody (AlexaFluor conjugate, Life Technologies, 1/1,000) and 300 nM 4′,6‐diamidino‐2‐phenylindole for 1 hour.

### Two‐Dimensional‐PAGE

Nuclear samples extracted from ESCs treated with LIF, or 1–4 days treatment with N2B27, were enzymatically labeled using Click‐IT *O*‐GlcNAc labeling system (Life Technologies) according to the manufacturers instructions. Samples were chloroform‐methanol precipitated and resuspended in 7 M urea, 2 M thiourea, 4% CHAPS, 1% ASB‐14, and 0.5% ampholytes. Passive rehydration was performed overnight followed by isoelectric focusing using pH 3–10 NL IPG strips for 4,200 vh. Preceding equilibration, samples were separated by SDS‐PAGE using 4%–12% bis/tris gels and transferred to Polyvinylidene fluoride for detection by streptavidin‐HRP. Ten prominent spots were excised from duplicate gels and analyzed by MALDI‐TOF mass spectrometry.

### Quantitative RT‐PCR

RNA was extracted from cells grown in six‐well plates using Nucleospin II RNA kit (Macherey‐Nagel, Dueren, Germany; www.mn-net.com). DNase treatment was performed on‐column during RNA extraction. One microgram of RNA was reverse transcribed with the qScript cDNA synthesis kit (Quanta Biosciences, Gaithersburg, MD; www.quantabio.com). PCR was performed on a BioRad iCycler or an AB 7500 using PerfeCTa SYBR Green FastMix for iQ or PerfeCTa SYBR Green FastMix Low Rox (both Quanta Biosciences), respectively. Primer sequences are shown in Supporting Information Table S1. Relative quantitation was performed using the method by Pfaffl 24 and β‐actin as a reference gene from technical duplicates or triplicates of at least three independent experiments.

### Microarray Analysis

RNA samples (from four independent experiments) were processed and hybridized to Affymetrix Gene 1.1ST arrays by Ark Genomics (www.ark‐genomics.org) and the raw array expression data were obtained as CEL files. For quality control and probe sets annotations, the annotations files (Release 32, dated 23‐06‐2011) downloaded from Affymetrix (Santa Clara, CA; www.affymetrix.com) were used. Background noise control (Detected Above Background), Robust Multi‐array Average normalization, and summarization of probe set level data into transcript clusters were carried out using Affymetrix Power Tools. Quality analysis and differential expression analyses were performed in Partek GS 6.5 (version 6.11.0321) software and R (version 2.13.1)—Bioconductor using Limma 25, and RankProd 26 packages 27. The microarray data have been deposited in EBI ArrayExpress under accession number E‐MEXP‐3593. For gene set enrichment analysis (GSEA) GSEA v2.0.13 was used, along with gene sets from MSigDB, GenesigDB, or custom made ones from the literature (Supporting Information Table S5). Genes were ranked by the Signal2Noise metric and the weighted2 enrichment statistic was used over 1,000 gene set permutations. A false discovery rate *q*‐value cut‐off of 0.05 was applied.

## Results

### Protein O‐GlcNAcylation Delays Mouse ESC Differentiation

**Figure 1:**
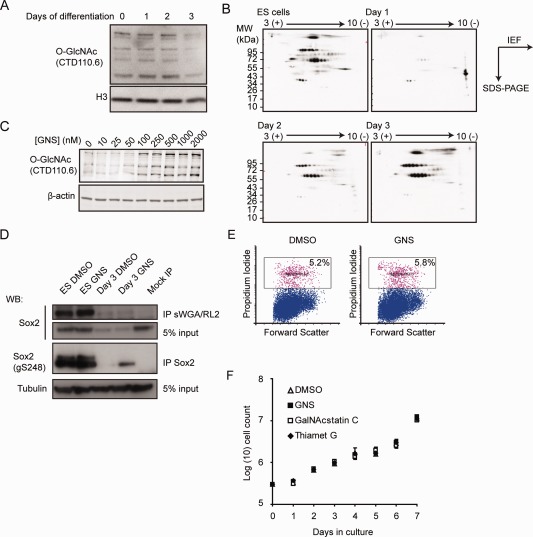
Global protein *O*‐GlcNAcylation in embryonic stem cells (ESCs). **(A):** Whole‐cell lysates of ESCs and cells at various stages of neural differentiation were blotted for *O*‐GlcNAc levels. No major changes are detectable in the most abundant bands. **(B):** Two‐dimensional gels of nuclear extracts blotted for *O*‐GlcNAcylation. The overall level of *O*‐GlcNAc is reduced in differentiation conditions whereas a cluster of basic proteins is prominent in the Day 1 sample. **(C):** Dose‐dependent increase in protein *O*‐GlcNAcylation after 24 hours treatment with 1 µM GNS. **(D):** Undifferentiated and 3‐day neural differentiated ESCs in DMSO or GNS were immunoprecipitated with antibody against *O*‐GlcNAc (RL‐2) and succinylated WGA beads or antibody against Sox2 then blotted for Sox2 or Sox2 GlcNAcylated at S248. GNS increases *O*‐GlcNAc levels on Sox2, especially on day 3. **(E):** Measurement of apoptotic cells (pink) after 24 hours of vehicle (DMSO) or 1 µM GNS treatment shows no difference in apoptosis levels. **(F):** ESC proliferation in vehicle (DMSO), the Oga inhibitors Thiamet G, and GNS or its inactive stereoisomer, GalNacstatin C (all at 1 µM). Abbreviations: GNS, GlcNAcstatin C; *O*‐GlcNAc, *O*‐linked *N*‐acetylglucosamine.

We decided to investigate the changes in *O*‐GlcNAc signaling during ESC neural differentiation. Global *O*‐GlcNAc levels decline slightly during the first few days of differentiation, as measured by Western blotting with the *O*‐GlcNAc‐specific antibody CTD110.6 (Fig. 1A). However, this method may miss changes in the *O*‐GlcNAcylation of lower abundance proteins. To test for more subtle changes in protein *O*‐GlcNAcylation, we performed a two‐dimensional‐PAGE‐Western blot analysis of nuclear extracts of ESCs and cells during the first 4 days of monolayer differentiation using chemoenzymatic labeling of *O*‐GlcNAcylated proteins (Fig. 1B and Supporting Information Fig. S1A). The results revealed a dynamic pattern of nuclear *O*‐GlcNAcylated proteins, with a dramatic change during the first 24 hours of differentiation. As the *O*‐GlcNAcylation on the majority of the nuclear proteins disappeared following 24 hours of differentiation, we hypothesized that removal of the *O*‐GlcNAc modification from some ESC protein(s) may be required for differentiation onset. We also picked 10 spots showing dramatic regulation from the Day 1 gel and identified them by mass spectrometry. Our analysis identified 26 unique proteins. Although we were not able to positively identify *O*‐GlcNAc modification on any of these proteins due to the instrumentation used, we note that 13 of the 26 had previously been identified as *O*‐GlcNAc modified by other screens (Supporting Information Table S2).

We then sought to determine whether the *O*‐GlcNAc levels in ESCs can be modulated by inhibiting the Oga enzyme activity that removes *O*‐GlcNAc using GNS, a highly potent and specific small molecule Oga inhibitor that exhibits 164‐fold selectivity for Oga when tested against the closely related lysosomal hexosaminidases HexA/B 28 and a competitive inhibitor of the *O*‐GlcNAc transferase Ogt (4Ac5SGlcNAc) 29. Treatment of ESCs with GNS resulted in a dose‐dependent increase in cellular *O*‐GlcNAc levels (Fig. 1C) including *O*‐GlcNAcylation of the transcription factor Sox2 on S248 (Fig. 1D), with no discernable effects on cell viability or proliferation even after prolonged treatment (Fig. 1E, 1F). Similar results were obtained by use of siRNA (Supporting Information Fig. S1B). Inhibition of Ogt conversely led to cell death within 4–5 days in culture (Supporting Information Fig. S1C), consistent with the previous observation that this protein is essential for ESC viability 5 (Fig. 3A).

**Figure 2:**
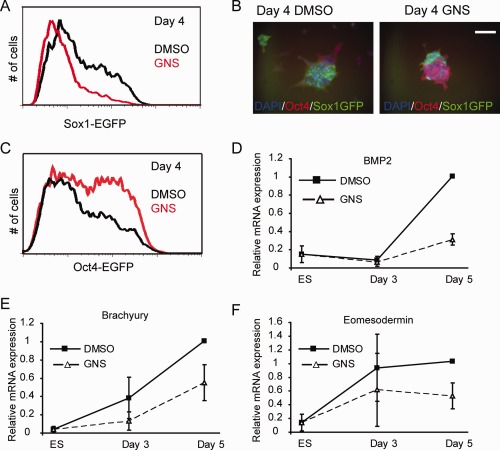
GlcNAcstatin treatment impairs embryonic stem cell (ESC) differentiation. **(A):** The proportion of Sox1GFP expressing neural progenitors is decreased following GNS treatment during differentiation of 46C cells. **(B):** 46C cells retain expression of Oct4 when differentiated in the presence of GNS while control cells differentiate into neural progenitors. Scale bar = 50 µm. **(C):** Oct4GiP cells retain Oct4‐GFP expression at higher levels in differentiation conditions compared to vehicle only controls. **(D–F):** ESCs were differentiated as embryoid bodies in the presence of DMSO or GNS for 3 or 5 days then assayed for expression of endodermal marker BMP2 (D) or mesodermal markers Brachyury (E) or Eomesodermin (F) by RT‐qPCR. Mean ± SEM from *n* = 3 experiments. Abbreviations: DAPI, 4′,6‐diamidino‐2‐phenylindole; GFP, green fluorescent protein; GNS, GlcNAcstatin C.

We then treated murine 46C ESCs (engineered to express the green fluorescent protein [GFP] from the endogenous neural‐specific *Sox1* locus 21) with GNS during monolayer differentiation into neural cells 20. Flow cytometry showed that a significantly smaller proportion of neural progenitors were generated during ESC differentiation in the presence of GNS (24.6% in GNS vs. 32.2% in DMSO at day 3, a reduction of 23.6%; *n* = 4, *p* = .0118) (Fig. 2A, Supporting Information Videos S1 and S2). Similar effects were seen using a structurally distinct Oga inhibitor, Thiamet G (21.4% reduction at day 3). *Sox1* mRNA levels were similarly reduced (Supporting Information Fig. S2A). This decrease in neural progenitors could reflect a bias of differentiation against neural fate, or be due to a more general effect on the onset of differentiation irrespective of lineage. To test this differentiating 46C cells were assessed for expression of the ESC marker Oct4. GNS‐treated ESCs remained Oct4 positive when vehicle‐treated control cells have largely lost Oct4 immunoreactivity and instead expressed Sox1GFP (Fig. 2B). We obtained the same result using the transgenic *Oct4*‐GFP reporter ESC line Oct4GiP (Fig. 2C), suggesting a delay in differentiation onset in the presence of GNS. Consistently, we also observed a decrease in endoderm (BMP2) and mesoderm (Brachyury, Eomesodermin) markers following EB differentiation (Fig. 2D–2F). Interestingly, primitive endoderm differentiation measured by RT‐qPCR for Sox17 and Gata6 appears to be unaffected by GNS (Supporting Information Fig. S2B, S2C).

**Figure 3:**
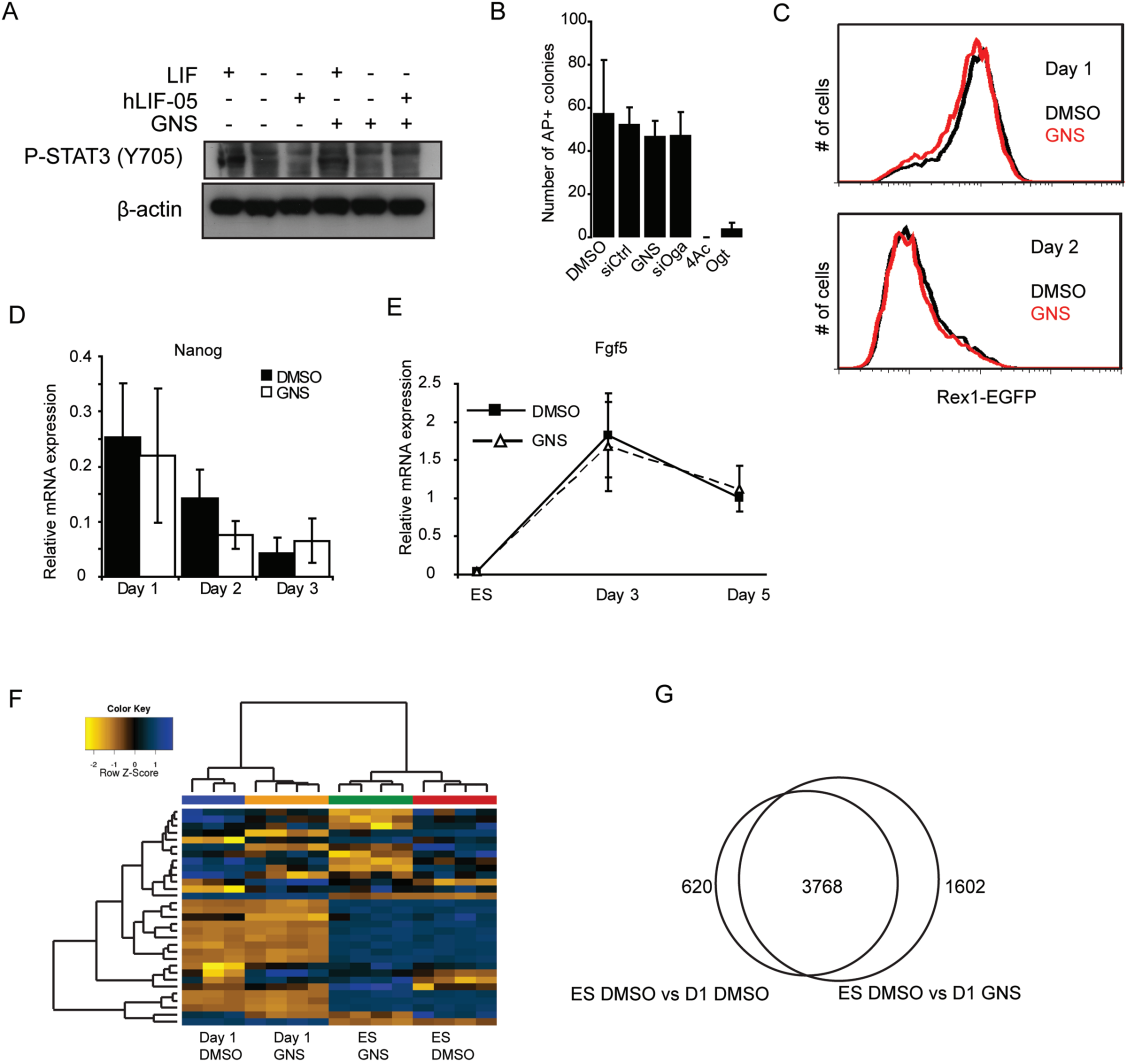
Increased *O*‐GlcNAcylation does not affect naïve‐primed embryonic stem cell (ESC) transition. **(A):** 46C ESCs were cultured in the absence of LIF overnight and then stimulated with either LIF or the LIF receptor antagonist hLIF‐05 in the presence of either DMSO or GNS, then blotted for phospho‐Stat3 (Y705). GNS treatment does not interfere with the ability of LIF to stimulate Stat3 phosphorylation and does not cause Stat3 phosphorylation in the absence of LIF. **(B):** GNS or Oga knockdown does not affect the ability of ESCs to form undifferentiated colonies, while inhibition or knockdown of Ogt results in cell death. 4Ac: 4‐Acetyl‐5S‐GlcNAc (Ogt inhibitor). **(C):** OCRG9 ESCs (Rex1EGFP reporter line) were differentiated in N2B27 for 2 days in vehicle or GNS and analyzed by flow cytometry for EGFP fluorescence. GlcNAcstatin treatment does not affect the loss of naïve marker Rex1. **(D):** Quantitative RT‐PCR analysis during monolayer differentiation shows that expression of the naïve marker Nanog is lost at equivalent rates in the presence of vehicle (DMSO; black bars) or GNS (white bars). **(E):** Upregulation of the primed pluripotency marker Fgf5 is unaffected by GNS treatment (D and E, *n* = 3, mean ± SEM). **(F):** Hierarchical clustering (using Euclidean distance with complete linkage agglomeration method) of samples based on the top 31 differentially expressed genes (ranked by *p*‐value) separates the samples according to the stage of differentiation and shows clustering of genes that contribute to differentiation. **(G):** Pairwise comparison of genes regulated during the first 24 hours of differentiation in vehicle (DMSO) or GNS (fold change >1.2, *p* < .05). Abbreviation: GNS, GlcNAcstatin C.

ESC self‐renewal can be maintained through the action of the transcription factor Stat3, operating downstream of the cytokine LIF. However, we could not detect any change in Stat3 phosphorylation in GNS‐treated cells compared to controls (Fig. 3A), which suggests that the effect of raised *O*‐GlcNAc levels on ESC differentiation is independent of Stat3 activity. Furthermore, GNS is not able to substitute for LIF and promotes the clonal expansion of ESCs in serum (Supporting Information Fig. S3A), nor does it affect cloning efficiency in the presence of LIF (Fig. 3B), consistent with a delay rather than a complete block in differentiation.

### *O*‐GlcNAc Levels Do Not Affect Transition of Naïve Cells to a Primed State

Recent work has suggested that *O*‐GlcNAcylation of Oct4 can maintain ESCs by regulation of downstream naïve state markers like *Nanog*, *Rex1*, *Klf2*, *Klf* *5* and others 11. When cultured in the presence of serum and LIF, ESC populations consist of mixed naïve and primed cells, but in serum‐free N2B27 monolayer differentiation conditions the transcripts for the naïve markers decline sharply after ∼24 hours and the cells proceed to differentiate. To test whether this early transition is affected by *O*‐GlcNAc levels we used the OCRG9 line, expressing GFP under the control of the endogenous *Rex1* locus. Under control conditions, OCRG9 cells become GFP negative at day 2 of differentiation (the delay between loss of *Rex1* mRNA and loss of GFP is due to the stability of the latter). GNS‐treated OCRG9 cells lost expression of *Rex1*‐GFP at the same rate as the control (DMSO‐treated) cells (Fig. 3C). The transition from naïve to primed state is mediated by the actions of the Erk1/2 kinases downstream of fibroblast growth factor (Fgf) signaling. Recent work in *Drosophila* has identified a requirement for *O*‐GlcNAcylation for Erk1/2 signal transduction downstream of Fgf receptor 30. However, consistent with a normal transition to the primed state, we did not detect any changes in the profile of Erk1/2 phosphorylation in GNS‐treated cells (Supporting Information Fig. S3B), suggesting that the signal regulating transition of naïve cells to primed pluripotency is unaltered by increased *O*‐GlcNAc levels.

This is further confirmed by quantitative RT‐PCR for other naïve cell markers *Nanog*, *Rex1/Zfp42*, *Esrrb*, and *Dppa3* (Fig. 3D, Supporting Information Fig. S3C) as well as the transient upregulation of the epiblast marker Fgf5 in EB differentiation (Fig. 3E). Taken together, these results indicate that *O*‐GlcNAc levels do not affect the initial events of ESC differentiation.

In order to test this hypothesis further we performed a microarray experiment to measure GNS's effect on global transcriptional changes during the transition of naïve pluripotent cells to a primed state. We treated ESCs with DMSO or GNS for 24 hours either in ESC media or in neural differentiation conditions and compared gene expression between treatments. Principal component analysis showed that the change from self‐renewal media to monolayer differentiation media (including serum withdrawal) causes a major effect on global gene expression (37%). This is reflected in the clustering of the most significantly regulated genes that change their expression from ESCs to day 1 of differentiation irrespective of GNS (Fig. 3F). We then compared the changes in gene expression during the first 24 hours of differentiation in the presence of GNS or DMSO compared to undifferentiated ESCs. This revealed that a great majority of the genes are common to the two conditions (Fig. 3G), consistent with the hypothesis that naïve‐to‐primed transition is unaffected by *O*‐GlcNAc levels. Many of the genes downregulated (fold change >1.2, *p* < .05) in both vehicle and GNS samples belong to pathways regulating ESC self‐renewal (i.e., MAPK, Jak/Stat and Tgfβ superfamily; Table 1) and include *Bmp4*, *Tgfβ1* and downstream targets *Id1*, *Id2*, *Id3*; *Stat3* and downstream target *Socs3* as well as transcription factors associated with naïve pluripotency (*Klf2*, *3*, *4*, and *5*, *Nanog*, *Rex1/Zfp42*). Common upregulated genes include neural and mesodermal early differentiation regulators (*Otx2*, *Tbx4*, *Neurogenin3*, *NeuroD1*, and *NeuroD4*) as well as primed pluripotency epiblast marker *Fgf5*, consistent with our RT‐qPCR results. In total, 45 genes from our set also belong to the Plurinet network of pluripotency‐related markers 31, 20 of which are upregulated in ESCs, and 25 are upregulated in the day 1 samples (Supporting Information Tables S3 and S4, respectively). This result confirms that raised *O*‐GlcNAc levels do not affect the expression of genes controlling the onset of differentiation in serum‐free monolayer.

**Table 1:** KEGG pathway analysis of the genes significantly regulated during the first 24 hours of embryonic stem cell differentiation irrespective of GlcNAcstatin C treatment

Regulation	Term	Count	% of genes in group	*p*‐Value	Benjamini
Up in ES	Focal adhesion	57	0.3	2.30E‐12	3.90E‐10
Up in ES	Lysosome	39	0.2	1.90E‐10	1.60E‐08
Up in ES	ECM‐receptor interaction	28	0.2	5.70E‐08	3.10E‐06
Up in ES	Regulation of actin cytoskeleton	45	0.2	1.70E‐05	7.20E‐04
Up in ES	TGF‐beta signaling pathway	24	0.1	2.80E‐05	9.20E‐04
Up in ES	Pathways in cancer	59	0.3	3.90E‐05	1.10E‐03
Up in ES	Tight junction	31	0.2	6.70E‐05	1.60E‐03
Up in ES	Leukocyte transendothelial migration	28	0.2	1.10E‐04	2.20E‐03
Up in ES	MAPK signaling pathway	49	0.3	1.50E‐04	2.70E‐03
Up in ES	ARVC	20	0.1	2.50E‐04	4.20E‐03
Up in ES	Small cell lung cancer	21	0.1	5.00E‐04	7.50E‐03
Up in ES	Other glycan degradation	8	<0.1	7.50E‐04	1.00E‐02
Up in ES	Glycosphingolipid biosynthesis	7	<0.1	2.10E‐03	2.60E‐02
Up in ES	Endocytosis	36	0.2	2.60E‐03	3.00E‐02
Up in ES	Jak‐STAT signaling pathway	29	0.2	2.70E‐03	3.00E‐02
Up in ES	HCM	19	0.1	2.90E‐03	3.00E‐02
Up in ES	Glycosphingolipid biosynthesis	7	<0.1	3.20E‐03	3.10E‐02
Up in ES	ErbB signaling pathway	19	0.1	4.30E‐03	3.90E‐02
Up in ES	Amino sugar and nucleotide sugar metabolism	12	0.1	5.50E‐03	4.70E‐02
Up in ES	Galactose metabolism	9	<0.1	5.80E‐03	4.70E‐02
Up in ES	Dorso‐ventral axis formation	8	<0.1	6.30E‐03	4.90E‐02
Up in Day 1	Steroid biosynthesis	11	0.1	1.90E‐08	3.40E‐06
Up in Day 1	Terpenoid backbone biosynthesis	8	0.1	1.30E‐05	1.10E‐03
Up in Day 1	Biosynthesis of unsaturated fatty acids	9	0.1	2.60E‐04	1.50E‐02
Up in Day 1	Pathways in cancer	38	0.3	6.90E‐04	3.00E‐02

Abbreviations: ARVC, arrhythmogenic right ventricular cardiomyopathy; HCM, hypertrophic cardiomyopathy.

### Increased Protein *O*‐GlcNAcylation Does Not Interfere With Phosphorylation of Major Signaling Pathways in ESCs

*O*‐GlcNAc is frequently attached to serine and threonine residues that can also be phosphorylated and for many proteins this reciprocal relationship (often described as a “Yin‐Yang”) regulates their activity. To investigate whether increased *O*‐GlcNAc levels affect the global phosphorylation of proteins in ESCs we compared the global phospho‐serine and phospho‐threonine levels of ESC protein extracts treated with various concentrations of GNS. Unlike global *O*‐GlcNAc levels that showed a dose‐dependent increase, protein phosphorylation levels remained relatively constant, suggesting that the majority of ESC phosphosites are not occupied by *O*‐GlcNAc (Supporting Information Fig. S4A). This result, however, could reflect a high abundance of constitutively phosphorylated proteins in these cells that conceal significant changes in less abundant and more dynamically regulated phosphoproteins. We therefore focused our attention on protein kinases as these proteins are often dynamically regulated by phosphorylation and mediate numerous important cellular responses. We analyzed kinase phosphorylation in whole‐cell lysates from ESCs treated for 24 hours with either DMSO or GNS using a protein kinase array. However, none of the 46 kinase sites profiled showed a significant change in their basal level of phosphorylation by GNS (Supporting Information Fig. S4B) indicating that the ESC kinome is not significantly affected by the Yin‐Yang interplay between *O*‐GlcNAc and phosphate.

### Raised *O*‐GlcNAc Levels Affect Transcription of Repressed Genes in ESCs

**Figure 4:**
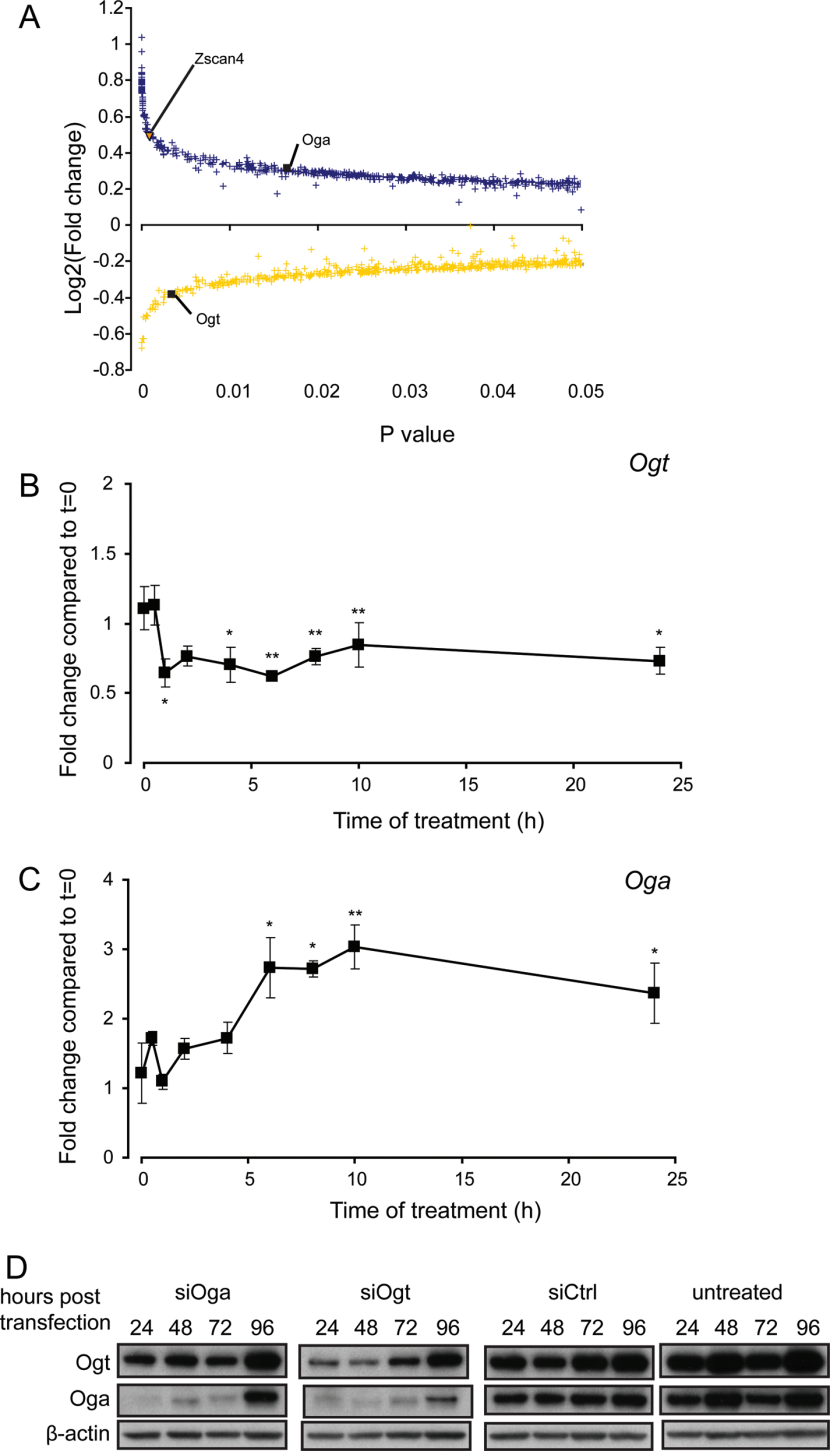
*O*‐linked *N*‐acetylglucosamine levels control Ogt and Oga levels in embryonic stem cells (ESCs). **(A):** Ranked product analysis of genes regulated in ESCs by GlcNAcstatin C treatment. Blue: significantly upregulated genes; yellow: significantly downregulated genes. **(B):** Validation of *Ogt* regulation by RT‐qPCR (*n* = 4, mean ± SEM). **(C):** Validation of *Oga* expression by RT‐qPCR (*n* = 4, mean ± SEM). **(D):** RNAi knockdown of Ogt and Oga results in a negative feedback regulation on the expression of Oga and Ogt, respectively. *, *p* < .05; **, *p* < .01 compared to *t* = 0 (*t* test).

We then analyzed ESCs treated for 24 hours with DMSO or GNS by microarrays, using Ranked Product analysis—a method better at detecting changes in gene expression from small number of replicates than the more commonly used Significance Analysis of Microarrays or Analysis of Variance methods 32. Using a Ranked Product *p*‐value < .05 we identified 971 differentially regulated genes. Of these, 516 were upregulated following GNS treatment and 455 were downregulated. Two of the most significantly regulated genes are *Ogt* and *Oga* (Fig. 4A). Using RT‐qPCR we found that *Ogt* expression is significantly reduced within 1 hour of treatment with GNS, whereas *Oga* upregulation appears slower (Fig. 4B, 4C). The regulation of Oga and Ogt by *O*‐GlcNAc levels is also reflected at the protein level within 24 hours of treatment with GNS (Supporting Information Fig. S4C) or by knockdown of Ogt or Oga using siRNA (Fig. 4D).

**Figure 5:**
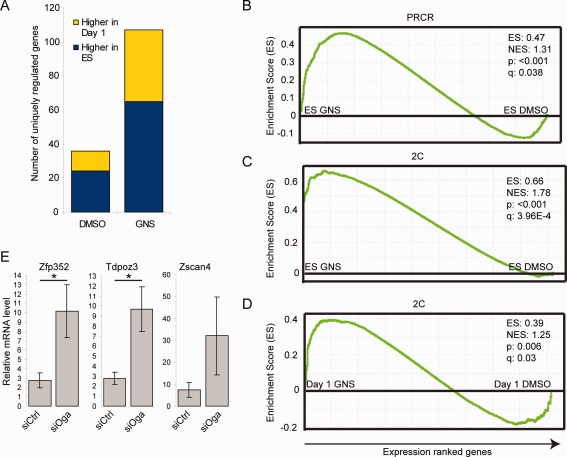
Oga inhibition results in a broad genomic transcriptional derepression. **(A):** Bar charts showing the number of unique genes upregulated in ESCs or in Day 1 differentiation in the DMSO or GNS samples (*p* < .05, fold change >2). **(B):** Gene set enrichment analysis (GSEA) shows that GNS treatment results in an upregulation of the “Polycomb repressed” gene set. **(C):** GSEA showing enrichment of the 2C gene set in the GNS‐treated ESCs. **(D):** The 2C gene set is also enriched in the GNS‐treated Day 1 differentiating cells. **(E):** RT‐qPCR validation of the upregulation of three genes from the 2C cohort by knockdown of Oga (*n* = 3, mean ± SEM) *, *p* < .05. Abbreviation: GNS, GlcNAcstatin C.

One other gene that stood out from this analysis is Zscan4 (Fig. 4A). This gene has previously been associated with telomere maintenance in ESCs 33 and is associated with a subpopulation of cells similar to that of the recently described “2C” state of privileged developmental plasticity, existing within ESC cultures 34, 35, 36. 2C cells differ in gene expression from ESCs in that they express genes associated with zygotic genome activation and have been demonstrated to be totipotent (giving rise to extraembryonic as well as embryonic tissues in chimeras) 36. Transcripts marking this subpopulation include retrotransposons normally repressed by epigenetic mechanisms as well as chimeric transcripts of genes with junctions to murine endogenous retrovirus with leucine tRNA primer (MERVL) elements 36. Interestingly, the number of genes upregulated in the GNS‐treated samples was much larger than the DMSO samples, both for the genes enriched in ESCs and for genes enriched in Day 1 differentiating cells. We therefore performed pairwise comparisons between the ESC and Day 1 samples in DMSO or GNS treatment for the most regulated genes (*p* < .05, fold change >2). The majority of genes with expression higher in ESCs than Day 1 (∼80%; 348/437) were similarly regulated both in DMSO and GNS. However, of the remaining 20% (those that were not common to GNS and DMSO), nearly three times more genes were higher in the GNS sample than in DMSO (65 vs. 24; Fig. 5A). Similarly, the majority of the genes expressed at higher level in Day 1 samples compared to ESCs are common to GNS and DMSO (∼75%; 160/214), but of those differentially regulated between the treatments, those regulated by GNS outnumbered those regulated by DMSO by a factor of 3.5 (42 vs. 12; Fig. 5A). This result suggests that GNS treatment causes a general increase in gene expression both in ESCs and early differentiating cells (Fig. 3G). We then turned to GSEA for further mining of our expression data. Search of the whole MSigDB and GeneSigDB databases using GSEA did not reveal a significant enrichment for any gene set so we created our own gene sets from publicly available microarray and ChIP‐seq data as published in relevant papers. We focused our attention to targets of the ESC core pluripotency network 37, 38 and genes regulated by the Polycomb group 39, 40, 41. Although there was no significant enrichment for polycomb complex 1 or 2 targets in our dataset (Supporting Information Table S6), a specific subset of polycomb target genes, designated as Polycomb‐repressed (PRCR) 39, is highly significantly enriched in ESCs treated with GNS compared to the vehicle control (Fig. 5B, Table 2). Strikingly, we also detected highly significant enrichment in the GNS‐treated samples for the gene sets corresponding to the 2C population (the 2C sets as described previously 36) as well as the gene set “2C‐MERVL+” which comprises the genes from the 2C set that contain MERVL chimeric transcripts. The enrichment of 2C transcripts is highly significant both in undifferentiated ESCs and in Day 1 differentiation samples treated with GNS compared to their respective vehicle controls (Fig. 5C, 5D, Table 2, Supporting Information Tables S6 and S7). We validated the upregulation of three 2C associated genes (Zfp352, Tdpoz3, and Zscan4) by Oga knockdown and GNS in ESCs using quantitative RT‐PCR and show a consistent upregulation for all three (Fig. 5C, Supporting Information Fig. S5A–S5C). These results therefore indicate that increased *O*‐GlcNAc levels result in increased expression of epigenetically repressed genes, including those characteristic of a totipotent 2C state.

**Table 2:** Gene sets significantly enriched in GlcNAcstatin C or DMSO‐treated embryonic stem cell or Day 1 differentiation samples

Name	Size	Enrichment score	Normalized enrichment score	Nominal *p*‐value	False discovery rate *q*‐value	Enrichment in
2C‐MERVL+	45	0.85	1.85	<.001	<0.001	ES GNS versus ES DMSO
2C	466	0.66	1.78	<.001	0.001	ES GNS versus ES DMSO
PRCR	1,331	0.47	1.31	<.001	0.038	ES GNS versus ES DMSO
2C	466	0.39	1.25	.006	0.033	Day 1 GNS versus Day 1 DMSO

## Discussion

Our results demonstrate that ESC differentiation is reduced under conditions of increased *O*‐GlcNAc signaling and pinpoint the deficit to a stage in differentiation after the loss of naive pluripotency but before the definitive differentiation as marked by the loss of Oct4 expression. Using a range of differentiation protocols we found a delay in the onset of differentiation toward embryonic lineages. We report that Oga inhibition does not affect the upregulation or downregulation of the genes known to be regulated early during differentiation, but results in prolonged expression of the pluripotency master regulator Oct4 and a delay in the acquisition of stable neural differentiation marker Sox1. Therefore, our results clearly demonstrate that Oga inhibition does not affect the transition from the naïve to the primed state, indicating instead that elevated *O*‐GlcNAc levels affect differentiation progression at a later stage. Taken together, our data show a clear effect of Oga inhibition on the initiation of ESC differentiation that affects multiple somatic lineages.

We also report that Oga inhibition does not interfere with the steady‐state phosphorylation levels of several key kinases, suggesting that the main mechanism of action operates downstream of the signal transduction machinery. The fact that the phosphorylation status of kinases is unaffected by GNS in this context does not, however, preclude changes in kinase activity, localization, or interaction with substrates. It also remains possible that the stimulated, maximal phosphorylation levels of some of these kinases will be affected by elevated *O*‐GlcNAc despite no obvious effects at the basal phosphorylation levels.

An early study of Oga inhibition during ESC differentiation identified a deficit in spontaneous cardiomyocyte generation in EBs 12. In that study a different, less specific Oga inhibitor was added to 5‐day‐old differentiating EBs, when ESC markers are already lost and the cells have already committed to differentiating, highlighting a role for Oga in the commitment of mesodermal cells to the cardiomyocyte lineage. Our data extend and refine this previous observation by determining an earlier role in the exit from the pluripotent state. More recently, a report demonstrated a role for the *O*‐GlcNAc modification of Oct4 in the onset of ESC differentiation 11. Our results are consistent with those findings, although we find that naïve ESC markers decline normally under elevated *O*‐GlcNAc conditions, demonstrating that the earliest events in ESC differentiation are not *O*‐GlcNAc dependent.

A number of recent papers have linked the *O*‐GlcNAc transferase to the regulation of epigenetic regulators 16, 17, 18, 42. Our gene expression analysis has revealed that following Oga inhibition a number of repressed genes become activated. Gene set enrichment analysis showed a significant enrichment for a specific subset of polycomb repressor complex target genes, as well as genes associated with the early zygotic genome activation and retrovirus‐like elements. This latter gene expression signature is thought to mark a developmentally privileged ESC subpopulation capable of differentiation into extraembryonic lineages 35, 36. These genes are under the control of epigenetic regulators of gene expression and their expression can be induced by interfering with histone acetylation and methylation, either pharmacologically or by genetic deletion of key enzymes 36. Our findings therefore suggest that the levels of *O*‐GlcNAc in cells influence global gene expression regulated by major epigenetic mechanisms such as histone modifications and DNA methylation, and are consistent with a differentiation delay observed in ESCs treated with an inhibitor of histone deacetylases 43. Intriguingly, Oga has been shown to possess histone acetyltransferase (HAT) activity in vitro 44 and this activity is distinct from its GlcNAcase function. It is therefore likely that although inhibition of Oga's *O*‐GlcNAcase activity by GNS raises *O*‐GlcNAc levels and leads to feedback Oga upregulation, it does not affect the protein's HAT activity (as has been shown for the less specific inhibitor Streptozotocin 44). This could possibly lead to increased histone acetylation and the derepression of transcription we observe, although the observation that 2C genes were upregulated even when Oga protein levels are reduced by siRNA (Fig. 5E) indicates that this is not the main mechanism in this case. The negative feedback regulation from increased *O*‐GlcNAc levels to a reduction in Ogt protein levels may also contribute to the phenotypes observed, by disrupting the formation of some of its protein complexes. *O*‐GlcNAc can itself modify histones 45 and promote transcriptional activation 46, so the precise mechanism by which gene expression is regulated by *O*‐GlcNAc levels is likely to be complex and involve many, possibly redundant molecular players.

## Conclusions

In conclusion, our results demonstrate that ESC differentiation is delayed under conditions of increased *O*‐GlcNAc signaling and pinpoint the delay to a stage in differentiation after the loss of naive pluripotency but before commitment to differentiation as marked by the loss of Oct4 expression. We used defined media and short timescales to minimize the possibility of multiple effects on several interacting cell types, and found a measurable, significant defect in the progression toward differentiation. We attribute this differentiation defect to a disruption of the normal function of global activation and repression complexes, such as the histone deacetylases, polycomb group, and Ten‐eleven translocation proteins as well as direct effects of Oga and Ogt on chromatin.

## Author Contributions

C.M.S., T.C.E.D., and W.W.: collection and assembly of data and data analysis; K.S.: experimental design, collection and assembly of data, and data analysis and interpretation; M.M. and G.J.B.: data analysis and interpretation; D.M.F.v.A.: provision of reagents, experimental design, and data analysis and interpretation; M.P.S.: conception and design, data collection and assembly of data, data analysis and interpretation, manuscript writing, and final approval of manuscript.

## Disclosure of Potential Conflicts of Interest

The authors indicate no potential conflicts of interest.

## Supporting information

Supporting Information Table 1

Supporting Information Table 2

Supporting Information Table 3

Supporting Information Table 4

Supporting Information Table 5

Supporting Information Table 6

Supporting Information Table 7

Supporting Information Table 8

Supporting Information Figures

Supporting Information Video 1

Supporting Information Video 2
