# Supplementary material for: Elevated O‐GlcNAc Levels Activate Epigenetically Repressed Genes and Delay Mouse ESC Differentiation Without Affecting Naïve to Primed Cell Transition
Source: Stem Cells. 2014 Sep 15;32(10):2605–15. doi: 10.1002/stem.1761 (PMC4737245; doi:10.1002/stem.1761)
Supplement: Supplementary file 1 — Supporting Information Table 1 [file STEM-32-2605-s001.doc]

Supplementary Table 1

The primer sequences for the qRT-PCR experiments described in this study

| **Gene name** | **Forward primer (5’-3’)** | **Reverse Primer (5’-3’)** | **Primer Bank ID** |
| --- | --- | --- | --- |
| β-actin | GGCTGTATTCCCCTCCATCG | CCAGTTGGTAACAATGCCATGT | 6671509a1 |
| Bmp2 | gggacccgctgtcttctagt | tcaactcaaattcgctgaggac | 6680794a1 |
| Brachyury | gcttcaaggagctaactaacgag | ccagcaagaaagagtacatggc | 6678203a1 |
| Dppa3 | GACCCAATGAAGGACCCTGAA | GCTTGACACCGGGGTTTAG | 21218416a1 |
| Eomesodermin | ggcccctatggctcaaattcc | cctgccctgtttggtgatg | 258645095c1 |
| Esrrb | AACCGAAATGTCGTCCGAAGAC | GTGGCTGAGGGCATCAATC | 226958366c1 |
| Fgf5 | aagtagcgcgacgttttcttc | ctggaaactgctatgttccgag | 3721900a1 |
| Gata6 | TTGCTCCGGTAACAGCAGTG | GTGGTCGCTTGTGTAGAAGGA | 33859556a1 |
| Nanog | ATGCCTGCAGTTTTTCATCC | ACAGTCCGCATCTTCTGCTT | - |
| Oga | GGGTTATGGAGCAGAGAAAAGAG | CCTGGCGAAATAGCATAGATGAA | 15011884a1 |
| Ogt | GACGCAACCAAACTTTGCAGT | TCAAGGGTGACAGCCTTTTCA | 20982829a1 |
| Sox1 | TCGCTGCCACAGCACTACCA | TGCCTCCTCTGCGGACGATA | - |
| Sox17 | GATGCGGGATACGCCAGTG | CCACCACCTCGCCTTTCAC | 146134353c1 |
| Sox7 | atgctgggaaagtcatggaag | cgtgttctggtcacgagaga | 6755612a1 |
| Tdpoz3 | TCTCCAATGTCCAATGCTTTCTG | ACGGTTCCAACTATGCTCACC | 157057187c1 |
| Zfp352 | AAGTCCCACATCTGAAGAAACAC | GGGTATGAGGATTCACCCACA | 23821036a1 |
| Zfp42 | CCCTCGACAGACTGACCCTAA | TCGGGGCTAATCTCACTTTCAT | 7110739a1 |
| Zscan4 | CAGATGCCAGTAGACACCAC | GTAGATGTTCCTTGACTTGC | - |
