# Supplementary material for: Elevated O‐GlcNAc Levels Activate Epigenetically Repressed Genes and Delay Mouse ESC Differentiation Without Affecting Naïve to Primed Cell Transition
Source: Stem Cells. 2014 Sep 15;32(10):2605–15. doi: 10.1002/stem.1761 (PMC4737245; doi:10.1002/stem.1761)
Supplement: Supplementary file 3 — Supporting Information Table 3 [file STEM-32-2605-s003.doc]

Supplementary Table 3

Plurinet genes with a higher expression in ES cells compared to day 1 differentiation in both DMSO and GlcNAcstatin treated samples

| **Gene name** | **Regulation** |
| --- | --- |
| Acta1 | Up in ES |
| Anxa2 | Up in ES |
| Anxa3 | Up in ES |
| Apoe | Up in ES |
| Eppk1 | Up in ES |
| Erbb3 | Up in ES |
| Hist1h2bc | Up in ES |
| Mbd2 | Up in ES |
| Myc | Up in ES |
| Nanog | Up in ES |
| Phb | Up in ES |
| Plscr1 | Up in ES |
| Pnp | Up in ES |
| Ppap2c | Up in ES |
| Pxn | Up in ES |
| Rmnd5b | Up in ES |
| Rnd1 | Up in ES |
| Tdgf1 | Up in ES |
| Vamp8 | Up in ES |
| Zfp42 | Up in ES |
