# Supplementary material for: Elevated O‐GlcNAc Levels Activate Epigenetically Repressed Genes and Delay Mouse ESC Differentiation Without Affecting Naïve to Primed Cell Transition
Source: Stem Cells. 2014 Sep 15;32(10):2605–15. doi: 10.1002/stem.1761 (PMC4737245; doi:10.1002/stem.1761)
Supplement: Supplementary file 4 — Supporting Information Table 4 [file STEM-32-2605-s004.doc]

Supplementary Table 4

Plurinet genes with a higher expression in day 1 differentiation compared to ES cells in both DMSO and GlcNAcstatin treated samples

| **Gene name** | **Regulation** |
| --- | --- |
| Arid3b | Up in Day 1 |
| Casp9 | Up in Day 1 |
| Dhcr24 | Up in Day 1 |
| Dnmt3b | Up in Day 1 |
| Dscc1 | Up in Day 1 |
| Fam136a | Up in Day 1 |
| Fbp1 | Up in Day 1 |
| Gar1 | Up in Day 1 |
| Hmgb1 | Up in Day 1 |
| Msh3 | Up in Day 1 |
| Mutyh | Up in Day 1 |
| Myb | Up in Day 1 |
| Otx2 | Up in Day 1 |
| Pak1 | Up in Day 1 |
| Pak3 | Up in Day 1 |
| Pbx1 | Up in Day 1 |
| Pmaip1 | Up in Day 1 |
| Psip1 | Up in Day 1 |
| Rasl11b | Up in Day 1 |
| Rnmtl1 | Up in Day 1 |
| Rpa3 | Up in Day 1 |
| Sephs1 | Up in Day 1 |
| Snrpn | Up in Day 1 |
| Strbp | Up in Day 1 |
| Supt3h | Up in Day 1 |
