# Supplementary material for: Elevated O‐GlcNAc Levels Activate Epigenetically Repressed Genes and Delay Mouse ESC Differentiation Without Affecting Naïve to Primed Cell Transition
Source: Stem Cells. 2014 Sep 15;32(10):2605–15. doi: 10.1002/stem.1761 (PMC4737245; doi:10.1002/stem.1761)
Supplement: Supplementary file 9 — Supporting Information Figures [file STEM-32-2605-s009.pdf]

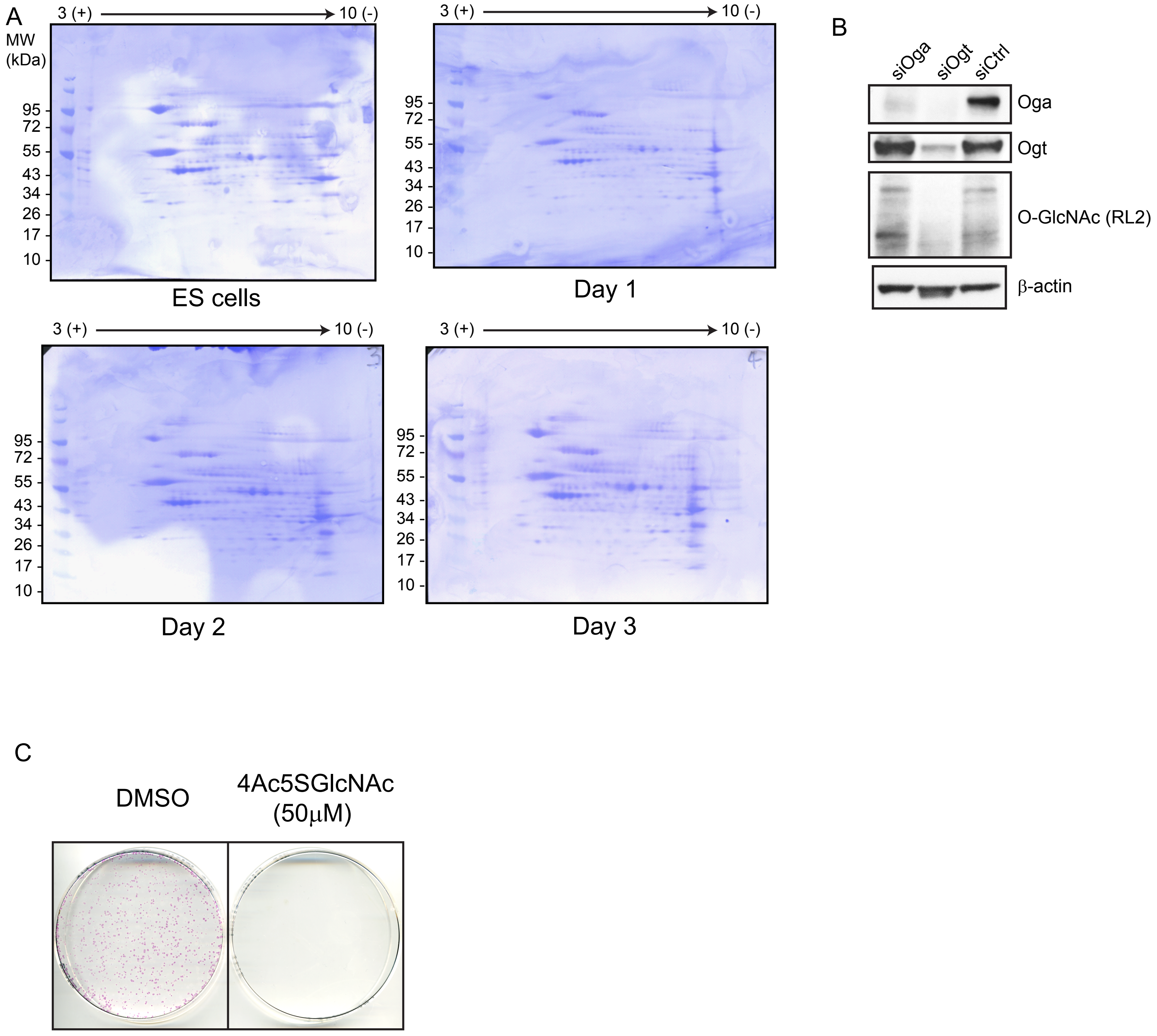

Stavridis Supplementary Figure 1

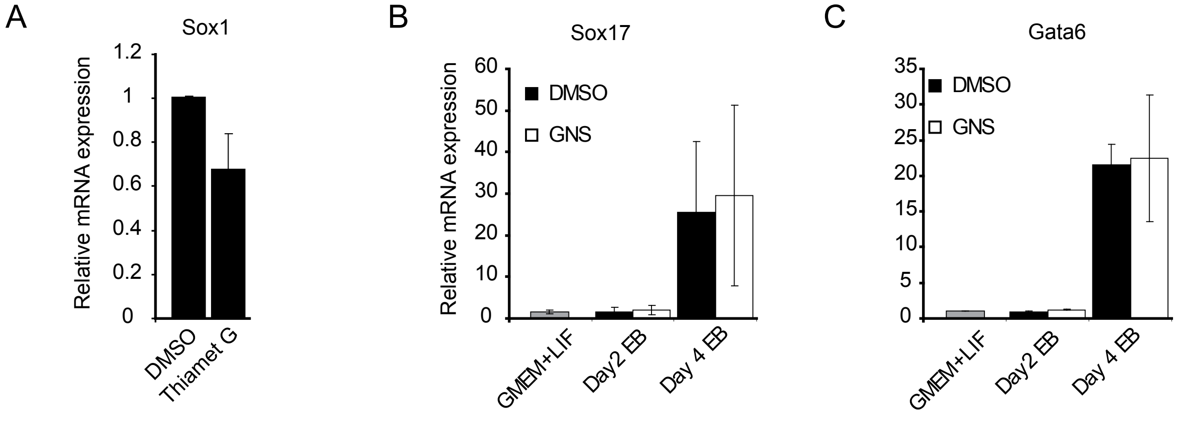

Stavridis Supplementary Figure 2

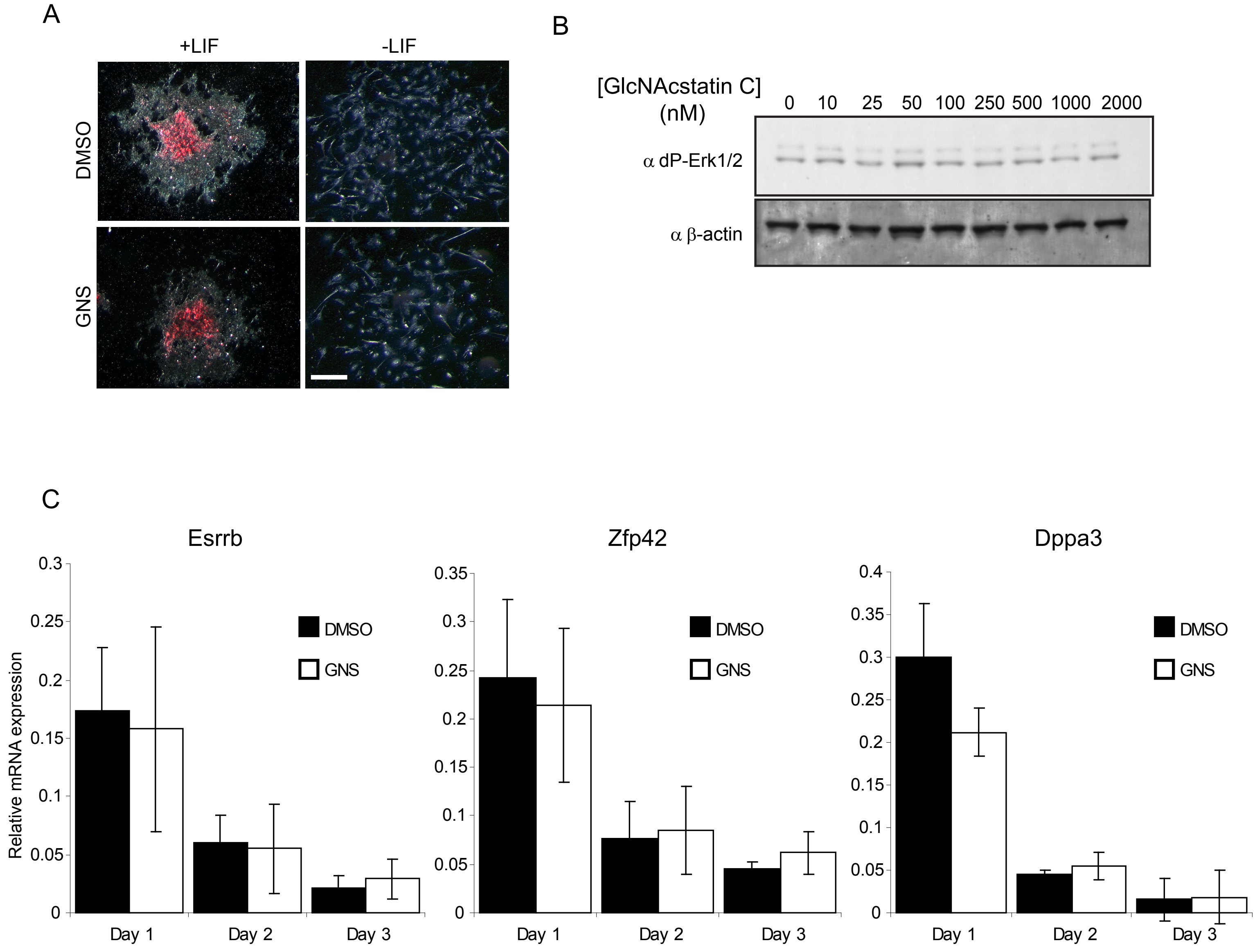

Stavridis Supplementary Figure 3

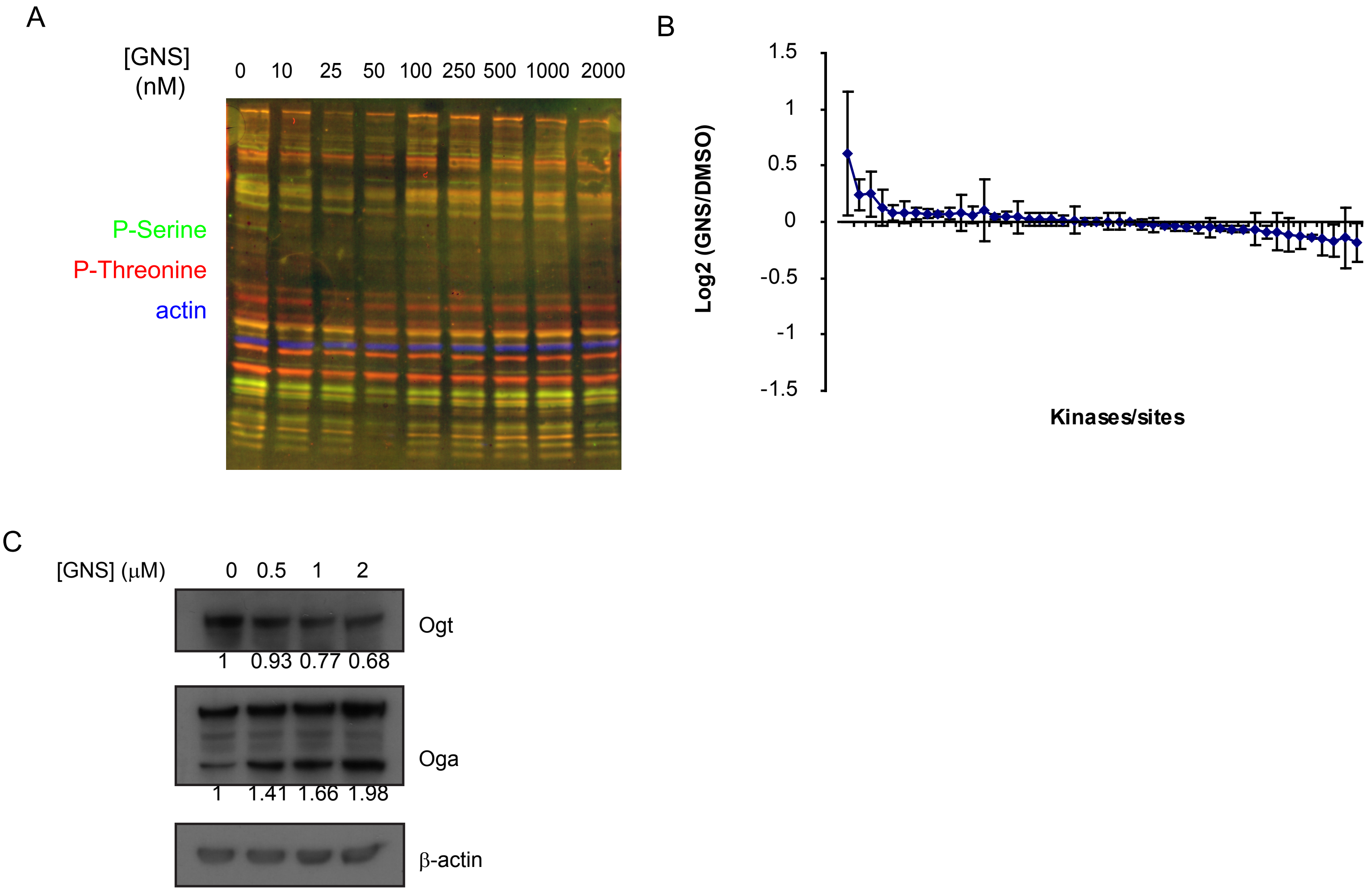

Stavridis Supplementary Figure 4

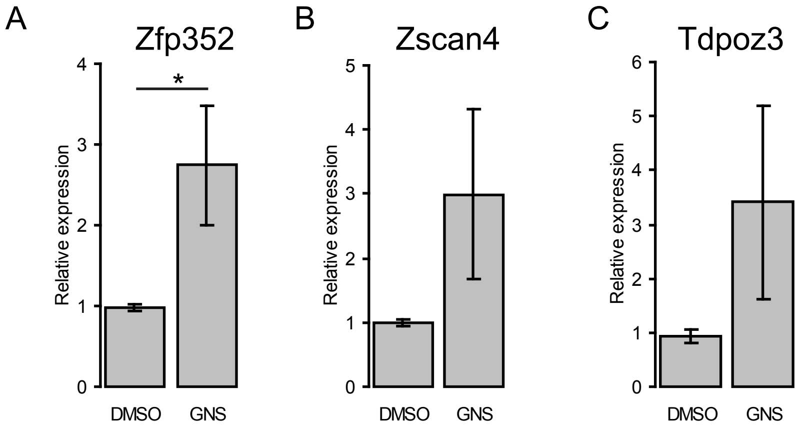

Stavridis Supplementary Figure 5

Stavridis Supplementary Figure 5
